# Efficacy and safety of aticaprant, a kappa receptor antagonist, adjunctive to oral SSRI/SNRI antidepressant in major depressive disorder: results of a phase 2 randomized, double-blind, placebo-controlled study

**DOI:** 10.1038/s41386-024-01862-x

**Published:** 2024-04-22

**Authors:** Mark E. Schmidt, Iva Kezic, Vanina Popova, Rama Melkote, Peter Van Der Ark, Darrel J. Pemberton, Guy Mareels, Carla M. Canuso, Maurizio Fava, Wayne C. Drevets

**Affiliations:** 1 Janssen Research & Development, Beerse, Belgium; 2 Janssen Research & Development, LLC, Titusville, NJ USA; 3 Massachusetts General Hospital and Harvard Medical School, Boston, MA USA; 4 Janssen Research & Development, LLC, San Diego, CA USA

**Keywords:** Drug development, Psychiatric disorders

## Abstract

This was a double-blind, randomized, phase 2 study of adults (18–64 years) with *DSM−5* diagnosis of major depressive disorder (MDD), with moderate-to-severe episode severity (Montgomery–Åsberg Depression Rating Scale [MADRS] ≥25) despite an adequate course with ongoing antidepressant for ≥6 weeks to ≤12 months. Following a double-blind placebo lead-in period (up to 3 weeks), participants were randomized to receive once daily aticaprant 10 mg or continue placebo, added to their ongoing treatment, for 6 weeks. Of 184 participants enrolled, 169 were included in safety analyses (aticaprant *n* = 85, placebo *n* = 84) and 166 in full intent-to-treat (fITT) efficacy analyses; 121 placebo lead-in non-responders (<30% reduction in MADRS total score) in fITT were included in enriched ITT (eITT) analyses. Improvement (least squares mean difference [upper limit 1-sided 80% CI] versus placebo) in MADRS total score at week 6 for aticaprant was significant versus placebo (eITT: −2.1 [−1.09], 1-sided *p* = 0.044; effect size (ES) 0.23; fITT −3.1 [2.21], 1-sided *p* = 0.002; ES 0.36). The between-group difference was larger among participants with Snaith–Hamilton Pleasure Scale (SHAPS) score greater/equal to versus less than baseline median SHAPS. The most common treatment-emergent adverse events reported for aticaprant (versus placebo) were headache (11.8% versus 7.1%), diarrhea (8.2% versus 2.4%), nasopharyngitis (5.9% versus 2.4%), and pruritus (5.9% versus 0%). One participant (1.2%) in each arm discontinued treatment due to an adverse event. In this study of participants with MDD and inadequate response to SSRI/SNRI, adjunctive treatment with aticaprant significantly reduced depressive symptoms versus placebo, without resulting in significant safety signals, supporting further investigation in larger trials.

## Introduction

Major depressive disorder (MDD) is a leading cause of disability, affecting approximately 280 million individuals worldwide [1]. MDD is associated with increased mortality and risk for suicide [1, 2]. As previously described [3], anhedonia, the lack of reactivity to pleasurable stimuli, is a cardinal feature of MDD and has received renewed interest as a potential endophenotype of this debilitating disease [4].

Inadequate response to first-line standard-of-care antidepressant treatment for MDD remains a significant problem [5–7], leaving many patients with substantial, persistent impairment [8]. While switching antidepressants and using adjunctive treatments may improve response, almost 40% of patients remain symptomatic and fail to achieve remission despite multiple treatment trials [9, 10]. During treatment the persistence of core depressive symptoms, including anhedonia, leads to longer time to remission, reduces the chance of achieving full recovery, and increases the vulnerability of MDD patients to future depressive episodes [11–14]. Furthermore, reward processing deficits in patients with MDD contribute to functional impairment, which putatively contribute to persistent and prolonged anhedonia [15]. Additional treatments, targeting this core depressive symptom, are needed for patients with partial or no response to current antidepressants [14].

MDD has been associated with dysregulation of the endogenous mu- and kappa-opioid systems [16, 17]. Kappa opioid receptors (KOR) and the endogenous peptide ligand dynorphin are expressed in brain regions/circuits involved with stress and reward and may play a key role in the pathophysiology of mood, stress, reward mechanisms, and addictive disorders [16, 18, 19]. Emerging evidence implicates KOR system overactivation in mediating depressive symptoms [20], especially anhedonia [21]. KOR antagonism has been tested in preclinical models of depression and anhedonia, and found to have meaningful effects that may translate to therapeutic benefit in humans with mood disorders, especially by modulating the negative affective state associated with responses to stress [16, 22].

We conducted a phase 2 study of aticaprant, a high-affinity, selective kappa receptor antagonist (KRA), with the aim of determining its efficacy and safety as an adjunctive therapy in patients with MDD. This clinical development study follows on single- and multi-dose phase 1 studies of aticaprant [23] and a positron emission tomography scan occupancy study which identified a clinical dose of 10 mg as achieving saturation of brain KOR at maximal concentrations, while providing a suitable safety margin [19]. Furthermore, in the FAST-fail trial in Mood and Anxiety Spectrum disorders (FAST-MAS), a proof-of-mechanism study of participants with anhedonia and a history of MDD or anxiety disorder, treatment with aticaprant 10 mg daily for 8 weeks increased activity in the ventral striatum during a reward anticipation task, improved reward learning, and reduced anhedonic symptoms assessed by the Snaith–Hamilton Pleasure Scale (SHAPS) [24, 25]. In the current study, we tested the hypothesis that this aticaprant dose, administered adjunctively to a monoamine reuptake inhibiting antidepressant (to which participants with MDD had partially responded despite remaining moderate-to-severely depressed), would produce antidepressant effects assessed by change from baseline in the Montgomery–Åsberg Depression Rating Scale [26] (MADRS) score. We further tested a secondary hypothesis that aticaprant’s antidepressant effect would be greater in the subgroup with more severe anhedonia symptoms at baseline, assessed using the SHAPS as a surrogate marker for the impaired reward processing thought to reflect elevated dynorphin-kappa receptor signaling. Thus, the participants were stratified at randomization by baseline SHAPS score.

## Patients and methods

### Ethical practices

An independent review board (United States [US]) or ethics committee (ex-US sites) at each study site approved the study protocol and its amendments. The study was conducted in accordance with ethical principles that originated in the Declaration of Helsinki, current guidelines on Good Clinical Practices, and applicable regulatory and country-specific requirements. All individuals voluntarily provided written informed consent before participating in the study.

### Study design

This phase 2, multicenter (28 sites in the US, 11 in Russia, 5 in the United Kingdom, 6 in Ukraine, 2 in Germany, 1 in Moldova), randomized, double-blind, placebo-controlled study (clinicaltrials.gov: NCT03559192) was conducted between July 2018 and May 2020. The study consisted of a 5-week screening phase and an 11-week double-blind treatment phase, the latter consisting of 3 periods: (1) a double-blind placebo lead-in period of variable duration (up to 3 weeks); (2) a 6-week double-blind treatment period; and (3) a withdrawal period during which (only) participants who completed double-blind treatment received placebo for the remaining time of the treatment phase. Investigators and participants were informed that the variable duration of treatment with placebo during the lead-in period could last from 0 to 3 weeks, and that participants could be randomly assigned to treatment with either aticaprant or continued placebo during the 6-week double-blind period.

### Study population

Study participants were between 18 and 64 years of age with a diagnosis of MDD per *DSM−5* criteria [27], without psychosis; the current episode was to be 18 months or shorter in duration. Eligible participants had been treated with a selective serotonin reuptake inhibitor (SSRI) or serotonin-norepinephrine reuptake inhibitor (SNRI), at an adequate dosage for at least 6 continuous weeks, but no more than 12 months for the current depressive episode of moderate-to-severe severity, and had inadequate response documented at screening (i.e., based on MADRS [26] total score ≥25).

Individuals who had failed (≤25% improvement based on the Massachusetts General Hospital Antidepressant Treatment Response Questionnaire [MGH-ATRQ] [28]) three or more antidepressants, including the current SSRI/SNRI, during the current episode of depression, despite adequate dose and duration (≥6 weeks), were not eligible for study participation. The study excluded individuals with potentially confounding psychiatric and general medical comorbidities. All inclusion and exclusion criteria are presented in the *Supplement* (Table S1).

### Randomization and double-blind study drug

Eligible participants were randomly assigned (1:1), based on a computer-generated randomization schedule, to receive double-blind treatment with either 10 mg aticaprant (JNJ−67953964) or to continue matching placebo, each once daily (in fasting condition, before breakfast) for 6 weeks during the double-blind treatment period. Randomization was balanced by using randomly permuted blocks of 4, stratified by placebo lead-in response status and SHAPS score (≥20 versus <20). Treatment codes were assigned by a centralized interactive web response system. Participants continued taking the same SSRI/SNRI antidepressant/dose that they had received prior to study entry, with no changes permitted during the study. The use of quetiapine (≤100 mg) was allowed, primarily as a sedative, when it had been used in a stable dose for ≥8 weeks prior to screening and was continued unchanged during the study. Participants, investigators/site personnel, those assessing outcomes, and those analyzing the data were blinded to treatment group assignment.

Adherence was tracked by the site, with study drug dispensing and return recorded in the electronic case report form. Adherence to study drug was also monitored using a smart phone app that recorded study drug ingestion.

### Efficacy assessments

Severity of depression was assessed by site-based, trained, certified, and blinded raters using the MADRS [26]. Severity of depressive illness was also assessed by investigators using the Clinical Global Impression–Severity (CGI-S; rated on a 7-point scale from 1 [normal – not at all ill] to 7 [among the most extremely ill patients]) [29].

Participants rated their hedonic capacity using the SHAPS, a reliable and validated 14-item instrument developed for use in MDD (score range 14–56; rating according Franken et al. [30]), with higher score indicating greater severity of anhedonia) [30, 31].

Investigators assessed anxiety using the Structured Interview Guide for the Hamilton Anxiety scale (SIGH-A; comprised of 14 items, each scored from 0 [not present] to 4 [maximum degree]) [32, 33] and the 6-item Hamilton Anxiety Subscale (HAM-A_6_; comprised of 5 psychic anxiety items [anxious mood, psychic tension, fears, intellectual disturbances, and anxious behavior] and 1 somatic item [muscular tension], each scored from 0 to 4) [34].

To assess the effect of study drug on aspects of cognitive and executive function, participants were asked to complete the Massachusetts General Hospital Cognitive and Physical Function Questionnaire (CPFQ) [35]. The CPFQ includes 7 questions about attention, energy, memory, mental acuity, and motivation, scored on a 6-point Likert scale, with higher values indicating worse function.

Samples were collected for exploratory plasma biomarkers and salivary cortisol; results will be reported elsewhere.

### Safety assessments

Adverse events and other standard safety assessments (i.e., hematology, serum chemistry, urinalysis, physical and neurological examination, vital signs, electrocardiogram [ECG]) were monitored throughout the study. Investigators classified adverse events as mild (i.e., easily tolerated, caused minimal discomfort and did not interfere with everyday activities), moderate (sufficient discomfort to cause interference with normal activity), or severe (i.e., caused extreme distress, significant impairment of functioning or incapacitation, and prevented normal everyday activities).

At each study visit, investigators administered the Columbia Suicide Severity Rating Scale (C-SSRS) questionnaire [36] to solicit the occurrence, severity, and frequency of suicide-related ideation and behaviors.

### Statistical methods

All randomized participants who received ≥1 dose of study drug during the double-blind treatment period were included in the safety analysis dataset, of whom those having ≥1 post-baseline MADRS assessment during the double-blind treatment period were included in a ‘full’ intent-to-treat (fITT) analysis dataset. Efficacy data were also analyzed for an “enriched” intent-to-treat (eITT) analysis dataset, which included all participants in the fITT analysis dataset who were non-responders at the end of the placebo lead-in period (i.e., <30% improvement in MADRS total score from screening or entry baseline). Investigators were blind to the definition of the improvement threshold. The 30% improvement threshold for detection of possible placebo response was selected based partly on a previous trial of ALKS−5461 (a combination of buprenorphine, a partial mu-opioid receptor agonist and KOR antagonist, and samidorphan, a potent mu-opioid receptor antagonist) [37], which used 50% improvement after 4 weeks of placebo (the first stage of their Sequential Parallel Comparison Design), and partly on our own adjustment for the shorter duration of the placebo lead-in, which was informed by a comparison of the prevalence of 30% and 50% improvement in participants assigned to placebo in previous Johnson & Johnson-sponsored placebo-controlled studies of other candidate antidepressants in MDD. The goal was to balance the response definition, while adjusting for the duration of the lead-in phase, and allowing for a typical attrition rate. The eITT analysis dataset was prespecified as the primary efficacy analysis dataset.

Statistical analyses were conducted using SAS, version 9.4. Analyses of efficacy endpoints were performed at a significance level of 0.20 (primary endpoint and other endpoints related to MADRS, 1-sided; secondary endpoints and other endpoints not pertaining to MADRS, 2-sided). Adjustment for multiple comparisons was not performed.

#### Efficacy endpoints and statistical analyses

The primary efficacy endpoint – change from treatment baseline to treatment week 6 in MADRS total score – was analyzed using a mixed-effects model using repeated measures (MMRM). The model included baseline MADRS score as a covariate; treatment (aticaprant, placebo), country, time, and time-by-treatment interaction as fixed effects; and a random patient effect.

In a pre-specified subgroup analysis, the impact of baseline anhedonia level (above versus below the baseline median SHAPS score) on the primary endpoint was summarized descriptively.

The overall differences between treatment groups based on the proportion of responders (defined by ≥30% and by ≥50% improvement from treatment baseline MADRS total score) and the proportion of participants in remission (MADRS ≤ 10) at the end of the 6-week double-blind treatment period were analyzed using Chi-square tests.

MADRS items most closely reflecting anhedonia symptoms (i.e., apparent sadness, reported sadness, concentration difficulties, inability to feel, and lassitude, referred to as the MADRS 5-item anhedonia factor subscale [38–40]; total score range 0–30) were examined *post hoc* according to the MMRM model described above for the primary efficacy endpoint analysis, but using the baseline MADRS 5-item anhedonia factor subscale score as covariate. Change in the MADRS 5-item anhedonia factor subscale score was also analyzed by baseline anhedonia level.

The same MMRM model, with respective baseline score as covariate, was also used to compare the treatment groups based on SHAPS score, SIGH-A total score, and HAM-A_6_ subscale score.

#### Analysis of safety endpoints

Treatment-emergent adverse events and other measures of safety were summarized descriptively for each treatment group.

#### Sample size determination

The sample size planned for this study was calculated assuming a treatment effect size of 0.45 at treatment week 6 for mean change from baseline in MADRS total score between aticaprant and placebo. The assumed effect size and an estimated standard deviation (SD) of 11 were derived from clinical trials of ALKS−5461 as adjunctive treatment in patients with MDD who had inadequate response to one or two courses of antidepressants [37, 41]. Based on an overall 1-sided significance level of 0.2 and SD of 11, randomization of about 90 individuals – 96 when adjusted for an anticipated 5% drop-out rate during the treatment period – was required to achieve 90% power. After adjusting for an estimated placebo response rate of 25% and drop-out rate of 10% during the placebo lead-in period, 142 participants were to enter the placebo lead-in period.

In accordance with the protocol, a blinded sample size re-estimation was conducted due to a higher-than-anticipated lead-in placebo response of 26.6% (Table S2), resulting in the inclusion of 181 participants.

The choice of alpha and beta (1-power) for this phase 2 study was intended to increase sensitivity for detecting a therapeutic signal while also maintaining a modest sample size. Thus, for the purpose of sample size estimation the power was set to a high value (power = 90%; beta = 0.10) but the type 1 error rate was specified at 1-sided alpha=0.20, as proposed by Lindborg and co-authors [42].

Consistent with the study design, the results for the analyses based on MADRS are characterized by 1-sided upper 80% CI, and those based on SHAPS by 2-sided 80% CI.

## Study results

Of 324 individuals screened, 181 were enrolled and entered the double-blind placebo lead-in period, of whom 169 were randomized and received ≥1 dose of study drug, and thus were included in the safety analysis dataset (Fig. S1). Three of these participants had no MADRS assessment during the treatment period, consequently 166 participants were included in the fITT analysis dataset, 124 (74.7%) of whom were placebo lead-in non-responders, while 42 (25.3%) were deemed placebo lead-in responders; 121 were included in the eITT analysis dataset. Of the 169 randomized participants, 161 (95.3%) completed double-blind treatment and entered the withdrawal period; 3 participants randomized to placebo (1 each due to adverse event, participant decision, and relocation) and 5 participants randomized to aticaprant (1 each due to adverse events [described in *Safety* section], lack of efficacy, noncompliance, protocol deviation, and self-imposed isolation during coronavirus pandemic) did not complete 6 weeks of double-blind treatment.

The mean (SD) exposure to placebo during the lead-in was 21.0 (1.31) days. Duration of the withdrawal period was 13.9 (1.88) days.

The treatment groups were similar with respect to demographic and baseline clinical characteristics (Table S2). As required for inclusion, all participants had been treated with an SSRI (131/169, 77.5%) or SNRI for the current episode prior to enrollment and continued them throughout the treatment period.

Adherence to study drug exceeded 90% for the trial, based on tracking in clinic or video recording via smart phone, and did not differ between the treatment groups.

### Efficacy

#### Change in depression severity

**Fig. 1:**
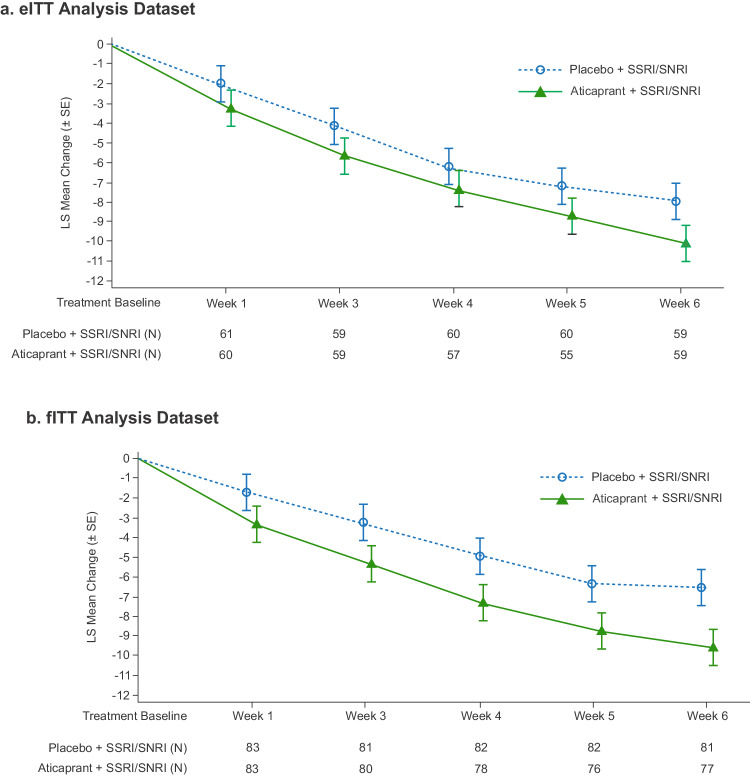
MADRS total score: LS mean change from baseline (±SE) over 6 weeks. eITT enriched intent-to treat, fITT full intent-to-treat, LS least squares, MADRS Montgomery–Åsberg Depression Rating Scale, SE standard error, SNRI serotonin-norepinephrine reuptake inhibitor, SSRI selective serotonin reuptake inhibitor. Note: MADRS total score ranges from 0 to 60; a higher score indicates a more severe condition. Negative change in score indicates improvement. Negative difference favors aticaprant.

In the eITT, prespecified primary efficacy analysis dataset, mean MADRS total score decreased from baseline to week 6, with greater improvement among those treated with aticaprant as compared to placebo, each adjunctive to ongoing antidepressant treatment (Table 1; least squares (LS) mean difference [upper limit 1-sided 80% CI]: −2.1 [−1.09], 1-sided *p* = 0.044; effect size (ES) 0.23). Improvement from baseline in MADRS score favored aticaprant over placebo at all time points during the 6-week double-blind treatment period, beginning at week 1 (eITT −1.2 [−0.19]; fITT −1.6 [−0.70]) and increasing with continuous dosing (Fig. 1a). The results of sensitivity analyses of the primary endpoint, conducted to evaluate the potential impact of COVID−19, are reported in the *Supplement*.

**Table 1:** MADRS total score: change from baseline to week 6 of double-blind treatment period.

	Aticaprant 10 mg + SSRI/SNRI	Placebo + SSRI/SNRI
eITT Analysis Dataset		
Baseline		
*N*	60	61
Mean (SD)	28.7 (3.58)	29.2 (5.47)
Change to week 6		
*N*	59	59
Mean (SD)	−10.2 (8.44)	−8.2 (8.53)
MMRM analysis^a^		
Difference of LS means (SE)	−2.1 (1.25)	
Upper limit 1-sided 80% CI on difference	−1.09	
1-sided *p* value	0.0443	
fITT Analysis Dataset		
Baseline		
*N*	83	83
Mean (SD)	24.8 (8.02)	25.7 (7.73)
Change to week 6		
*N*	77	81
Mean (SD)	−9.7 (8.02)	−6.6 (8.57)
MMRM analysis^a^		
Difference of LS means (SE)	−3.1 (1.05)	
Upper limit 1-sided 80% CI on difference	−2.21	
1-sided *p* value	0.0017	

*CI* confidence interval, *eITT* enriched intent-to-treat, *fITT* full intent-to-treat, *LS* least squares, *MADRS* Montgomery–Åsberg Depression Rating Scale, *MMRM* mixed-effect model using repeated measures, *SNRI* serotonin-norepinephrine reuptake inhibitors, *SSRI* selective serotonin reuptake inhibitors.

^a^MMRM analysis with change from baseline as the response variable, patient as a random effect, time, treatment, country, and time-by-treatment interaction as factors, and baseline value as a covariate.

Note: MADRS total score ranges from 0 to 60; a higher score indicates a more severe condition. Negative change in score indicates improvement. Negative difference favors aticaprant.

In the fITT analysis dataset, mean MADRS total score decreased from baseline to week 6, with greater improvement observed among those in the aticaprant group as compared with the placebo group (Table 1; LS mean difference [upper limit 1-sided 80% CI]: −3.1 [−2.21], 1-sided *p* = 0.002; ES 0.36) and over the 6-week treatment period (Fig. 1b).

**Fig. 2:**
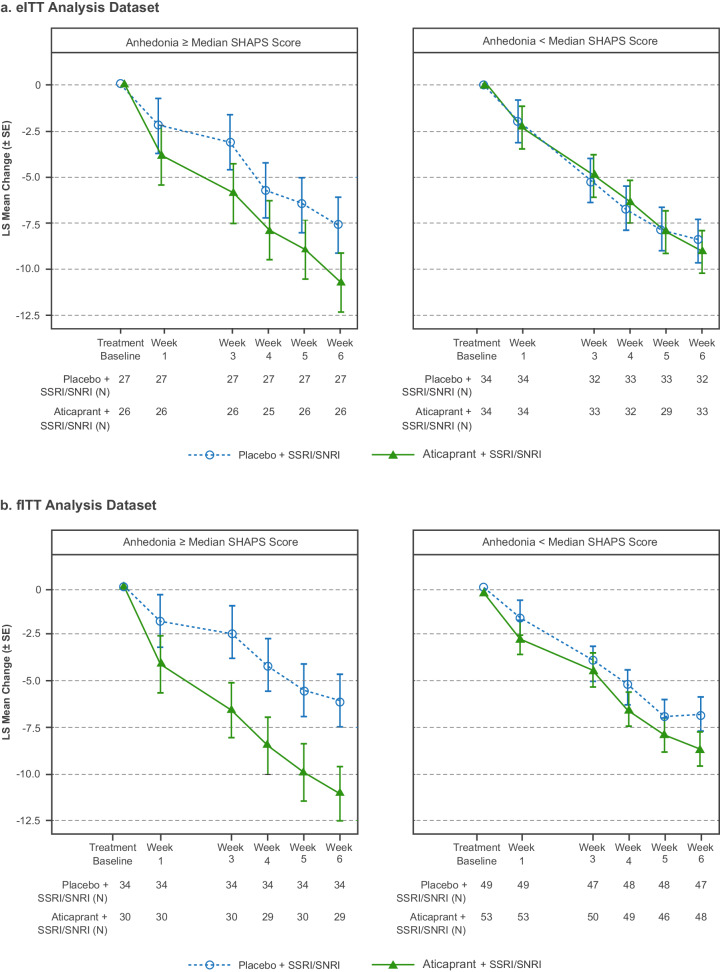
MADRS total score: LS mean change from baseline (±SE) over 6 weeks by level of anhedonia at baseline. eITT enriched intent-to treat, fITT full intent-to-treat, LS least squares, MADRS Montgomery–Åsberg Depression Rating Scale, SE standard error, SHAPS Snaith–Hamilton Pleasure Scale, SNRI serotonin-norepinephrine reuptake inhibitor, SSRI selective serotonin reuptake inhibitor. Note: MADRS total score ranges from 0 to 60; a higher score indicates a more severe condition. Negative change in score indicates improvement. Negative difference favors aticaprant.

In a subgroup analysis of the MADRS data, the between-group difference was larger among participants with high versus low anhedonia level (Fig. 2a [eITT] and Fig. 2b [fITT]), with the magnitude at week 6 (LS mean difference [upper limit 1-sided 80% CI]) smaller in the eITT versus fITT analysis dataset (high anhedonia level: eITT, −3.2 [−1.38], 1-sided *p* = 0.068, ES 0.36; fITT, −5.1 [−3.65], 1-sided *p* = 0.0015, ES 0.56; low anhedonia level: eITT, −0.6 [0.72], 1-sided *p* = 0.349, ES 0.07; fITT, −1.9 [−0.73], 1-sided *p* = 0.083, ES 0.24).

The proportion of participants who achieved remission and the proportion who were responders increased over time during the double-blind treatment period, to a greater extent in the aticaprant group than the placebo group (Figs. S2, S3). At week 6, the difference between groups was statistically significant, favoring aticaprant, for response rate in the fITT analysis dataset, but not for remission rate in either efficacy analysis dataset (Figs. S2, S3).

**Fig. 3:**
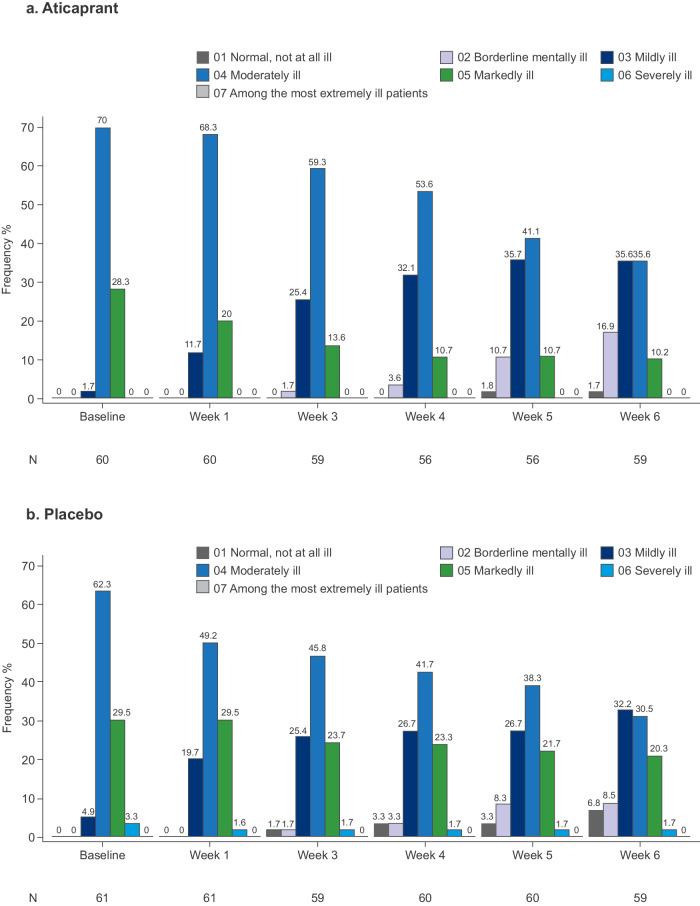
Frequency distribution of CGI-S score during the double-blind treatment period (eITT analysis dataset). CGI-S Clinical Global Impression—Severity, eITT enriched intent-to treat.

**Fig. 4:**
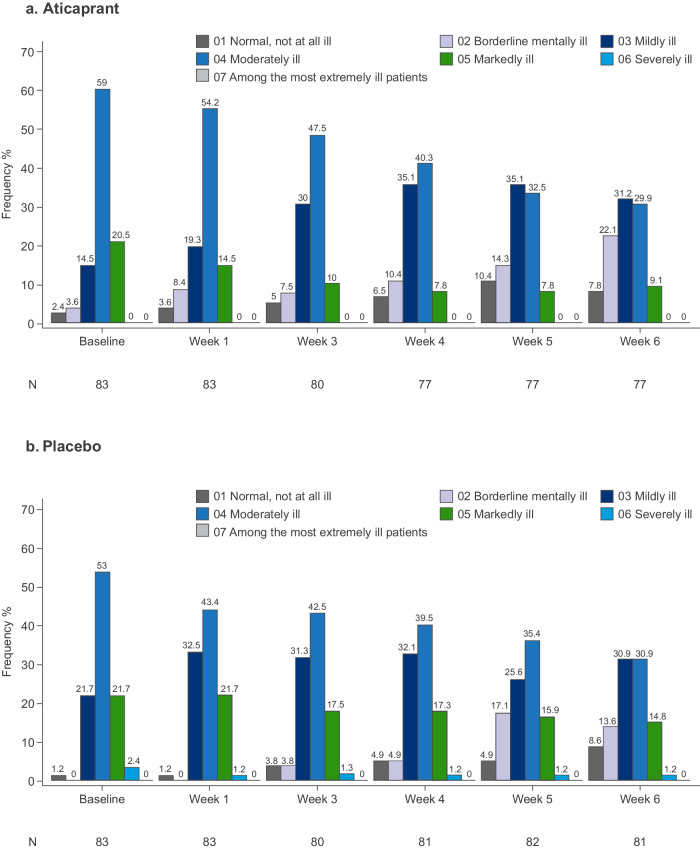
Frequency distribution of CGI-S score during the double-blind treatment period (fITT analysis dataset). CGI-S Clinical Global Impression—Severity, fITT full intent-to-treat.

Consistent with the MADRS assessments, mean CGI-S score numerically improved from baseline to week 6 (mean [SD]) in both the eITT analysis dataset (aticaprant: baseline 4.3 [0.48], change from baseline −0.9 [1.04]; placebo: baseline 4.3 [0.62], change from baseline −0.8 [0.86]) and in the fITT analysis dataset (aticaprant: baseline 3.9 [0.84], change from baseline −0.9 [1.01]; placebo: baseline 4.0 [0.80], change from baseline −0.7 [0.88]). The proportion of aticaprant-treated participants in the eITT analysis dataset with moderate or severe depressive illness (i.e., CGI-S score of ≥4) decreased progressively over the 6-week treatment period, to a numerically greater extent than among participants in the placebo group (Fig. 3). The same trend was observed in the fITT analysis dataset (Fig. 4).

#### Change in anhedonia severity

Mean (SD) change in SHAPS score from treatment baseline to week 6 was −4.6 (6.23) for aticaprant and −4.2 (5.04) for placebo in the eITT analysis dataset (Fig. S4a; LS mean between-group difference [2-sided 80% CI]: −0.7 [−1.81, 0.41], 2-sided *p* = 0.419; ES 0.07). Treatment effect was similar in the fITT analysis dataset (Fig. S4b; LS mean between-group difference [2-sided 80% CI]: −0.8 [−1.79, 0.10], 2-sided *p* = 0.250; ES 0.08).

In a *post hoc* analysis of anhedonia severity based on the SHAPS score, improvement in participant self-reported anhedonia was numerically greater with aticaprant compared to placebo (LS mean change [2-sided 80% CI]) in the subgroup of participants with higher baseline anhedonia severity (defined by SHAPS score greater/equal to baseline median) (eITT: −1.70 [−3.74, 0.34], 2-sided *p* = 0.284; fITT: −2.09 [−3.95, −0.22], 2-sided *p* = 0.152); improvement in severity of anhedonia was minimal in the subgroup of participants with lower baseline anhedonia severity and comparable between the treatment groups (eITT: 0.04 [−1.19, 1.27], 2-sided *p* = 0.966; fITT: −0.12 [−1.13, 0.89], 2-sided *p* = 0.878) (Table S3).

The prevalence of more severe anhedonia, characterized by SHAPS score greater than/equal to baseline median, was lower at week 6 than at baseline in both groups (eITT: aticaprant, 43.3% [baseline] versus 13.6% [week 6]; placebo, 44.3% versus 22.0%; fITT: aticaprant, 36.1% versus 10.4%; placebo, 41.0% versus 18.5%).

Results of *post hoc* analysis of anhedonia severity based on clinician-assessed 5-item MADRS anhedonia factor subscale score were consistent with those from participant-reported SHAPS score. Improvement in the 5-item MADRS anhedonia factor was greater with aticaprant compared to placebo (LS mean difference [upper limit 1-sided 80% CI]) among the participants with higher baseline anhedonia severity (eITT: −2.7 [−1.64], 1-sided *p* = 0.015; fITT: −3.6 [−2.59], 1-sided *p* = 0.001) and smaller for both treatment groups among the participants with lower baseline anhedonia severity (eITT: −0.4 [0.46], 1-sided *p* = 0.349; fITT: −1.2 [−0.57], 1-sided *p* = 0.059) (Table S4).

#### Change in anxiety severity

Mean SIGH-A total score decreased from baseline to week 6, with greater improvement of anxiety seen in the fITT analysis dataset (LS mean difference [80% CI] eITT dataset: −0.7 [−1.90, 0.41], 2-sided *p* = 0.410; fITT dataset: −1.4 [−2.31, −0.44], 2-sided *p* = 0.060) among participants treated with aticaprant compared to placebo, each adjunctive to SSRI/SNRI antidepressant. Consistent with these SIGH-A findings, treatment effect on anxiety at week 6 was also demonstrated based on HAM-A_6_ (eITT dataset: −0.6 [−1.21, 0.07], 2-sided *p* = 0.148; fITT dataset: −1.1 [−1.56, −0.62], 2-sided *p* = 0.003) (Table S5).

#### Change in cognition

Results of the CPFQ assessments are presented in the *Supplement*. The results indicate a numerically greater reduction in CPFQ total scores for aticaprant compared to placebo, although baseline to endpoint changes were not significantly different.

### Safety

#### Adverse events

During the placebo lead-in period, the only treatment-emergent adverse event reported in ≥5% of participants was headache (11/169, 6.5%) (Table S6).

During the double-blind treatment period, the most common treatment-emergent adverse events (incidence ≥5.0%) reported for aticaprant adjunctive to SSRI/SNRI were headache, diarrhea, nasopharyngitis, and pruritus (Table 2). Most events were of mild or moderate severity (248 of 251) and transient. No difference between treatment groups was observed in the incidence (2.4% each) of adverse events suggestive of abuse potential (defined in Table S7).

**Table 2:** Treatment-emergent adverse events in the double-blind treatment period.

	Number (%) of Participants	
	Aticaprant 10 mg + SSRI/SNRI *n* = 85	Placebo + SSRI/SNRI *n* = 84
Total Participants with AE	40 (47.1)	30 (35.7)
Adverse Events:		
Headache	10 (11.8)	6 (7.1)
Diarrhea	7 (8.2)	2 (2.4)
Nasopharyngitis	5 (5.9)	2 (2.4)
Pruritus	5 (5.9)	0
Nausea	4 (4.7)	2 (2.4)
Vomiting	4 (4.7)	1 (1.2)
Urinary tract infection	3 (3.5)	1 (1.2)
Contusion	2 (2.4)	0
Oropharyngeal pain	2 (2.4)	0
Somnolence	2 (2.4)	2 (2.4)

*SNRI* serotonin-norepinephrine reuptake inhibitor, *SSRI* selective serotonin reuptake inhibitor.

Note: The following adverse events were reported for 1 participant (1.2%) each in the aticaprant group: abdominal pain, anhedonia, blood creatine phosphokinase increased, back pain, bulimia nervosa colitis, costochondritis, dry skin, dyspnea, gastroenteritis viral, hordeolum, hyperesthesia, hyperhidrosis, hypoglycemia, influenza, insomnia, muscular weakness, oral herpes, presyncope, skin laceration, upper respiratory tract infection, vertigo.

Treatment-emergent adverse events reported for ≥1 participant in the placebo group and 0 in the aticaprant group are not presented.

During the withdrawal period, new-onset adverse events were reported for 5 (of 85, 5.9%) and 4 (of 84, 4.8%) participants who had been treated with aticaprant and placebo, respectively, in the double-blind treatment period (Table S8); none of these adverse events were considered by investigators to be related to withdrawal of study drug.

Serious treatment-emergent adverse events were reported for 2 participants: one during the placebo lead-in period (adverse event of suicidal ideation) and one in the placebo group during the double-blind treatment period (adverse event of acute cholecystitis). The latter participant and one other in the aticaprant group (adverse events of diarrhea, nausea, vomiting, and headache) discontinued the study drug prematurely due to adverse events. No deaths were reported in this study.

#### Suicidal ideation/behavior

A comparable proportion of participants in each treatment group reported suicidal ideation (i.e., C-SSRS score between 1 and 5) during the double-blind treatment period: 4.8% (4/83) and 3.6% (3/83) of participants in the aticaprant and placebo groups, respectively, at double-blind treatment endpoint. No participant in either treatment group experienced suicidal behavior in any study period.

#### Other safety assessments

There were no clinically meaningful changes in laboratory tests, vital signs, body weight (Table S9), or ECG. Results of laboratory testing, vital sign measurements, and ECG are summarized in the *Supplement*.

## Discussion

In this phase 2 study of aticaprant for patients with MDD who were inadequately responding to ongoing SSRI/SNRI antidepressants, adjunctive treatment with aticaprant led to statistically significantly greater reduction in depressive symptoms severity on the MADRS compared to placebo added to the ongoing antidepressant. The proportion of responders (≥30% and ≥50%) was also significantly higher with aticaprant. Remission rates did not differ to an extent that was statistically significant, although the difference in remission rates between aticaprant and placebo arms (31.2% and 22.2%, respectively) in the fITT analysis dataset was within the range commonly observed for clinical trials of approved antidepressants and adjunctive therapies for MDD. Moreover, a treatment period longer than 6 weeks may have been necessary to sensitively evaluate impact on remission [43]. The reliability of the data is supported by a low discontinuation rate during randomized treatment and high levels of adherence to treatment. Multiple approaches were used to encourage adherence, which may have enhanced participants’ engagement in the trial.

The greater treatment effect on the MADRS in the subgroup of participants with elevated anhedonia at baseline is noteworthy, suggesting that patients with MDD and more severe anhedonia may have greater benefit from adjunctive aticaprant. The greater effect of aticaprant in depressed participants with elevated anhedonia supports the hypothesized dysregulation of reward circuitry in depression and anhedonia [44]. Modulating dynorphin activity by a KRA putatively offers a means for restoring motivation and ability to experience pleasure in depression, as reflected previously by the mechanistic results of the FAST-MAS trial [24, 25] and herein, especially by improvement in MADRS anhedonia factor subscale scores.

The treatment effect in the fITT analysis dataset exceeded that in the eITT analysis dataset. A larger treatment effect and effect size had been predicted for the eITT analysis dataset that included only participants who were non-responders during the placebo lead-in period. However, among non-responders during the placebo lead-in period, the placebo response was 45.8%, showing that the lead-in period did not reduce the placebo response, which was slightly lower (44.4%) in the sample that included both responders and non-responders to the placebo lead-in period. Our findings are consistent with those of the placebo-controlled trials of aripiprazole in MDD, in which the MDD patients with MADRS below the median at the end of the lead-in phase had a greater effect size than the MDD patients with MADRS total score above the median [45]. The use of percent improvement from baseline has been used in previous trials [e.g., 37, 46] and was utilized in our trial at the end of the double-blind placebo lead-in phase. Notably, it did not include a minimum threshold score for severity at the end of the lead-in period (MADRS score ≥25 was required for inclusion). This could have resulted in excluding individuals from the eITT who achieved ≥30% improvement from baseline but still had at least moderate depression severity, and potential for further improvement. For example, a participant with MADRS total score of 35 and improvement of 30% would have a MADRS score of 24, reflecting moderate depression severity. Such an individual, who still had significant symptoms after the placebo lead-in and potential to respond, was excluded from the eITT yet included in the fITT analysis dataset.

In this study, improvement in participant-reported anhedonia, based on the SHAPS score, was observed in both treatment groups, although the between-group difference was not significant. In contrast, a significant treatment effect of aticaprant on the SHAPS was seen in the FAST-MAS trial [24, 25]. The divergent findings may be explained by the longer treatment period in the FAST-MAS trial, differences in severity of illness, or ongoing SSRI/SNRI use. The difference in anhedonia severity, rated by the SHAPS, was greater in the participants with higher baseline anhedonia severity, although the treatment difference was not significant. Moreover, analysis of anhedonia severity based on clinician-assessed 5-item MADRS anhedonia factor subscale score showed greater improvement with aticaprant compared to placebo among participants with higher baseline anhedonia severity in both the eITT and fITT datasets (Table S4).

Finally, the treatment effect on anxiety symptoms was greater in the fITT analysis dataset than in the eITT dataset as observed in the change in the HAM-A_6_ subscale and the full SIGH-A scale scores (Table S5).

In this study we tested the antidepressant efficacy of aticaprant administered adjunctively to SSRI/SNRI treatment to which participants had proven partially – but inadequately – responsive, evidenced by persistence of moderate-to-severe depressive symptoms. We hypothesized that the combination of the KRA and monoamine reuptake inhibitor mechanisms may exert synergistic effects on monoamine transmission [22]. The increased synthesis and release of dynorphin, induced under conditions of chronic stress or hypothalamic-pituitary-adrenal axis activation and some other physiological stressors, results in kappa receptor activation, which inhibits dopamine release from ventral tegmental area neurons during the processing of reward-related stimuli, putatively contributing to negative affective states and impaired reward learning [16, 17, 21]. Dynorphin-kappa receptor activation also reduces serotonin release from dorsal raphe nucleus projections to the nucleus accumbens, hippocampus, and other limbic regions in preclinical stress models, putatively contributing to anxiety and depression-like behaviors [17]. By blocking kappa receptor signaling, aticaprant may allow dopamine and serotonin release to return to adaptive levels during stress and reward processing, thereby producing antidepressant and anti-anhedonia effects. Crucially, by restoring normal release of monoamines, aticaprant may augment the efficacy of monoamine-reuptake inhibiting antidepressants, which can only increase synaptic levels of monoamine neurotransmitters after their release. Such a complementary effect of aticaprant to the effects of monoamine reuptake inhibiting antidepressants has been demonstrated in preclinical studies, which showed that antidepressant-like effects produced by concurrent administration of sub-active doses of aticaprant (3 mg/kg PO) and imipramine (5 mg/kg IP) were comparable with those produced by 15 mg/kg IP of imipramine [47, 48]. In these studies, synergy also was observed for the combination of aticaprant (3 mg/kg PO) and citalopram (3 mg/kg IP). While these data appear compatible with the clinical results reported herein, our study design cannot establish whether the antidepressant efficacy of aticaprant used adjunctively to monoamine reuptake inhibiting antidepressants in patients who previously had experienced inadequate antidepressant responses to such agents reflects a synergistic mechanism or only additive effects.

Safety results in this study were similar to the safety profile of aticaprant reported over an 8-week exposure period [24]. During the double-blind treatment period, the most common adverse events were headache, diarrhea, nasopharyngitis, and pruritus, each with incidence higher in the aticaprant group compared to the placebo group. Pruritus was previously reported in participants treated with aticaprant [24, 49]. Diarrhea was not reported in the FAST-MAS study with aticaprant monotherapy administered at 10 mg daily for 8 weeks [24] although it was observed in phase 1 studies of healthy volunteers at higher doses (described as ‘loose stools’ of mild severity which did not require treatment; unpublished data [NCT04185051]) and in persons diagnosed with cocaine dependence in early abstinence who received 10 mg daily for 4 days [49]. One (1.2%) participant in each group had adverse events leading to discontinuation of study drug, and one serious adverse event was reported (acute cholecystitis for a participant in the placebo group). Aticaprant may offer a more favorable safety profile compared to other adjunctive treatments to SSRI/SNRI antidepressants, and especially compared to approved adjunctive treatments for MDD, which currently are limited to atypical antipsychotic agents. Some of the latter agents commonly produce side effects such as weight gain, metabolic changes, extrapyramidal symptoms, or akathisia, none of which have been observed with aticaprant.

The generalizability of the study findings may be limited by the exclusion of participants with treatment-resistant depression, significant psychiatric co-morbidities, or substance dependence and by the preponderance of white study participants. This early-phase clinical trial aimed to assess the short-term efficacy and safety of aticaprant. Maintenance of antidepressant effect and long-term safety will be evaluated in phase 3 clinical development studies.

In conclusion, the favorable antidepressant effect and safety profile observed in this phase 2 study of aticaprant for patients with MDD and anhedonia, inadequately treated with SSRI/SNRI antidepressants, support further investigation of aticaprant in larger trials in MDD. Confirmatory trials of aticaprant as adjunct treatment for MDD and anhedonia are ongoing.

### Supplementary information

Supplementary Material

## Data Availability

The data sharing policy of Janssen Pharmaceutical Companies of Johnson & Johnson is available at https://www.janssen.com/clinical-trials/transparency. As noted on this site, requests for access to the study data can be submitted through Yale Open Data Access (YODA) Project site at http://yoda.yale.edu.
