# Supplementary material for: Efficacy and safety of aticaprant, a kappa receptor antagonist, adjunctive to oral SSRI/SNRI antidepressant in major depressive disorder: results of a phase 2 randomized, double-blind, placebo-controlled study
Source: Neuropsychopharmacology. 2024 Apr 22;49(9):1437–47. doi: 10.1038/s41386-024-01862-x (PMC11251157; doi:10.1038/s41386-024-01862-x)
Supplement: Supplementary file 1 — Supplementary Material [file 41386_2024_1862_MOESM1_ESM.docx]

**Contents of On-line Supplement**

Table S1. Patient Inclusion, Exclusion Criteria, and Lifestyle Considerations

Table S2. Summary of Demographic and Baseline Characteristics (Safety Dataset)

Figure S1. Disposition of Participants

Sensitivity Analyses of the Primary Endpoint

Figure S2. Percentage of Participants in Remission and Percentage Who Were Responders During the Double-Blind Treatment Period (eITT Analysis Dataset)

Figure S3. Percentage of Participants in Remission and Percentage Who Were Responders During the Double-Blind Treatment Period (fITT Analysis Dataset)

Figure S4. SHAPS Score: LS Mean Change from Baseline (± SE) During Double-Blind Treatment Period

Table S3. SHAPS Score: LS Mean Change from Baseline Over 6 Weeks by Level of Anhedonia Severity at Baseline

Table S4. MADRS 5-Item Anhedonia Factor Score: LS Mean Change from Baseline Over 6 Weeks by Level of Anhedonia Severity at Baseline

Table S5. SIGH-A Total Score and HAM-A_6_ Subscale Score: Change from Baseline to Week 6 of Double-Blind Treatment Phase

Massachusetts General Hospital Cognitive and Physical Function Questionnaire (CPFQ)

Table S6. Treatment-Emergent Adverse Events During the Placebo Lead-in Period

Table S7. Search of Preferred Terms to Identify Adverse Events Suggestive of Abuse Potential

Table S8. Treatment-Emergent Adverse Events During the Withdrawal Period

Other Safety Assessments

Table S9. Body Weight (kg): Mean Change from Baseline to Study Endpoint (Week 11)/Early Withdrawal

**Table S1. Patient Inclusion, Exclusion Criteria, and Lifestyle Considerations**

***Inclusion Criteria***

Each potential subject must satisfy all the following criteria to be enrolled in the study.

1. Subjects must be men or women, 18 to 64 years of age, inclusive.

Note: Subjects should be at least 18 years of age or older as per the legal age of consent in the jurisdiction in which the study is taking place.

1. Have a body mass index (BMI) between 18 and 35 kg/m^2^ inclusive (BMI = weight/height^2^).
2. Subjects must be medically stable based on clinical laboratory tests, medical history, vital signs, and 12-lead ECG performed at screening and baseline (Visit 2). For clinical laboratory tests only screening results will be considered. If the results of the serum chemistry panel, hematology, or urinalysis are outside the normal reference ranges, retesting of an abnormal lab value(s) that may lead to exclusion will be allowed once during the screening phase. In 12-lead ECG, QTcF should be ≤450 msec for males or ≤470 msec for females and PR-interval <220 msec at screening. A retest of an abnormal ECG value will be allowed once in the screening phase. Blood pressure will be the average of 2 measurements.

The subject may be included only if the investigator judges the abnormalities or deviations from normal to be not clinically significant or to be appropriate and reasonable for the population under study. This determination must be recorded in the subject's source documents and initialed by the investigator.

1. Population specific:
   - - Subjects must have a primary Diagnostic and Statistical Manual of Mental Disorders 5th edition (DSM-5) diagnosis of MDD. Subjects with a diagnosis of comorbid Generalized Anxiety Disorder (GAD), Social Anxiety Disorder (SAD), or Panic Disorder may be included, if the investigator considers MDD to be the primary diagnosis (confirmed by an independent central rater through review of the MINI interview obtained by the site at screening). The current episode should be less than 18 months.
     - Subjects must be currently treated with an SSRI/SNRI antidepressant approved in this protocol at an adequate dose, as defined by the ATRQ, and for at least 6 continuous weeks but not more than 12 months.
     - Have a MADRS total score of ≥25 at screening. If 2 weeks or more elapse between the MADRS rating at screening and Visit 2, the local rater will complete the MADRS again by a telephone interview up to 4 days before Visit 2. At this telephone interview, MADRS total score should again be ≥25 and should not demonstrate a clinically significant change (i.e., an improvement of >20%).
     - In a common population of subject suffering from MDD, about 90% have a SHAPS score >20. The SHAPS score will initially not be an inclusion criterion. However, after 50 subjects have been randomized, the subjects’ properties will be reviewed. If less than 50% of non-responding subjects have a SHAPS score >20, then for the remaining subjects the following inclusion criterion will be added: Have a SHAPS score >20 at screening and baseline (visit 2).
2. Men who are sexually active with a woman of childbearing potential and have not had a vasectomy must agree to use a barrier method of birth control i.e., a condom with spermicidal foam/gel/film/cream/suppository for the duration of the study plus 3 months after receiving the last dose of study drug, and all men must not donate sperm during the study and for 3 months after receiving the last dose of study drug. In addition, their female partners should also use an additional method of birth control (which may include a hormonal method, an intrauterine device [IUD] or an intrauterine system [IUS]) for at least the same duration.
3. Before randomization, a woman must be either:
   - - Not of childbearing potential defined as:
       - Postmenopausal (amenorrhea for at least 12 months without an alternative medical cause. A serum follicle stimulating hormone (FSH) level at screening >40 IU/L in women not using hormonal contraception or hormonal replacement therapy may be used for confirmation, however in the absence of 12 months of amenorrhea, a single FSH measurement is insufficient) or
       - Permanently sterilized (including hysterectomy, bilateral salpingectomy and bilateral oophorectomy) or
       - Otherwise be incapable of pregnancy.
     - Of childbearing potential and practicing a highly effective method of birth control consistent with local regulations regarding the use of birth control methods for subjects participating in clinical studies (i.e. one that results in a less than 1% per year failure rate when used consistently and correctly). This may include:
   - Established and ongoing use of oral hormonal methods of contraception in combination with barrier methods.
   - Established and ongoing use of patch, injected or implanted hormonal methods of contraception.
   - Placement of an IUD or IUS.

Accepted barrier methods as indicated above include:

- - - condom with spermicidal foam/gel/film/cream/suppository
    - occlusive cap (diaphragm or cervical/vault caps) with spermicidal foam/gel/film/cream/suppository.

Note that a barrier method on its own is not sufficient.

- - Male partner sterilization (the vasectomized partner should be the sole partner for that subject).
  - True abstinence from heterosexual intercourse (when this is in line with the preferred and usual lifestyle of the subject).

Women must agree to continue using these methods of contraception throughout the study and for at least 3 months after receiving the last dose of study medication.

Note: If a woman of childbearing potential who is not heterosexually active becomes active after the start of the study, she must begin a highly effective method of birth control, as described above.

1. A **woman** of childbearing potential must have a negative serum pregnancy test at screening and a negative urine pregnancy test before the first dose.
2. A **woman** must agree not to donate eggs (ova, oocytes) for the purposes of assisted reproduction during the study and for at least 3 months after receiving the last dose of study drug.
3. Subject must be willing and able to adhere to the prohibitions and restrictions specified in this protocol.
4. Sign an ICF indicating that they understand the purpose of and procedures required for the study including peripheral biomarkers research (i.e., blood) and are willing to participate in the study.

***Exclusion Criteria***

Any potential subject who meets any of the following criteria will be excluded from participating in the study. The subject will be excluded if he or she:

1. Has current signs/symptoms of, liver or renal insufficiency, hypothyroidism or hyperthyroidism (a normal thyroid-stimulating function is required at screening; subjects who are on stable treatment with thyroid supplementation with normal thyroid-stimulating hormone [TSH] may participate but subjects with thyroid supplementation for antidepressant purposes are not allowed in the study*), significant cardiac disease (including current or past history of atrial fibrillation/flutter), vascular, pulmonary, endocrine, neurologic (including epilepsy), hematologic, inflammatory (e.g., rheumatoid arthritis, inflammatory bowel disease, Crohn’s disease) or metabolic disturbances. Diabetes mellitus (DM) may be allowed when the subject is stable (HbA1c less than 7.5% or 58 mmol/mol).

*Subjects with known hypothyroidism who have been on stable treatment for at least 3 months prior to screening are required to have TSH and free thyroxine (FT4) obtained. Any subject with an elevated TSH should also have FT4 measured. In any case where the TSH value is out of range, but FT4 is normal, the findings should be discussed directly with the medical monitor before the subject is enrolled. If the FT4 value is out of range, the subject is not eligible.

1. History of documented gastric disease (including documented peptic ulcer disease, gastritis, upper GI bleeding, esophagitis, or any GI precancerous condition), current clinically evident GI complaints.
2. Chronic use of a proton pump inhibitors (PPIs). History of incidental use of PPIs is allowed but should have been stopped at least 4 weeks before screening. A history of chronic nonsteroidal anti-inflammatory drug (NSAID) or aspirin use. (Low dose aspirin e.g. in cardiovascular disease prevention is allowed).
3. Has a history of alcohol use disorder within the past year.
4. Has failed (no more than 25% response on ATRQ) three or more antidepressant treatments including the current SSRI/SNRI during the current depressive episode despite an adequate dose (per ATRQ) and duration (at least 6 weeks).
5. Subject has received an investigational drug (including investigational vaccines) or used an invasive investigational medical device within 3 months before the planned first dose of study drug, or has participated in any interventional clinical studies on MDD in the previous **1 year**, or is currently enrolled in an interventional study.
6. Has signs or symptoms of Cushing’s Disease, Addison’s Disease, primary amenorrhea, or other evidence of significant medical disorders of the HPA axis.
7. Is breast feeding.
8. Subject has a history of malignancy within 5 years before screening (exceptions are squamous and basal cell carcinomas of the skin and carcinoma in situ of the cervix, or malignancy that in the opinion of the investigator, with concurrence with the sponsor's study responsible physician, is considered cured with minimal risk of recurrence).
9. Has one or more of the following diagnoses:

- A primary DSM (5th edition) diagnosis of:
  - GAD
  - panic disorder
  - obsessive compulsive disorder (OCD)
  - posttraumatic stress disorder (PTSD)

Subjects with comorbid GAD, SAD, or panic disorder for whom MDD is considered the primary diagnosis are not excluded.

- A current diagnosis or diagnosis in the past 1 year of:
  - psychotic disorder
  - MDD with psychosis
  - anorexia nervosa or bulimia nervosa.
  - chronic fatigue syndrome
  - bipolar disorder (BD)
  - mental retardation
  - antisocial or borderline personality disorder
  - autism spectrum disorder.

1. Has a current or recent history of clinically significant suicidal ideation within the past 6 months, corresponding to a score of 4 (active suicidal ideation with some intent to act, without specific plan) or 5 (active suicidal ideation with specific plan and intent) for ideation on the C-SSRS, or a history of suicidal behavior within the past 1 year, as validated by the C-SSRS at screening or Visit 2. Subjects with a prior suicide attempt of any sort, or prior serious suicidal ideation/plan > 6 months ago, should be carefully screened for current suicidal ideation and only included at the discretion of the investigator.
2. Ongoing psychological treatments (e.g., Cognitive Behavior Therapy, Interpersonal Psychotherapy, Psychodynamic Psychotherapy etc.), initiated within 1 month prior to the screening phase. A subject who has been receiving ongoing psychological treatment for a period of greater than 1 month from the screening visit is eligible, if the investigator deems the psychological treatment to be of stable duration and frequency.
3. Subject has a history of hepatitis B surface antigen (HBsAg) or hepatitis C antibody (anti-HCV) positive, or other clinically active liver disease, or tests positive for HBsAg or anti-HCV at Screening. If subjects have been successfully treated for or have been spontaneously recovered from HCV and are RNA negative, they will be allowed in the study.
4. Subject has a history of human immunodeficiency virus (HIV) antibody positive, or tests positive for HIV at Screening. If subjects have been successfully treated for HIV and are ribonucleic acid (RNA) negative, they will be allowed in the study.
5. Subject has a history of substance use disorder according to DSM-5 criteria, except nicotine or caffeine, within 6 months before screening. Mild cases can be reviewed by investigator and study responsible physician on a case-by-case basis. Subjects who have completed a treatment for (alcohol) addiction more than 1 year prior to first dose administration, may be included if the risk of relapse is considered minimal, total duration of alcohol use disorder was less than a year, and no significant abnormalities are shown in clinical laboratory or other predose safety assessments.
6. Subject has positive test result(s) for alcohol or drugs of abuse (including barbiturates, methadone, opiates, cocaine, cannabinoids, amphetamine/methamphetamine, and ecstasy) at Screening or at baseline (visit 2). A positive test result for benzodiazepines is not exclusionary if the subject is taking such drugs per protocol.

Subjects with a positive alcohol or drug screen may have the test repeated once, based on the investigator's discretion. This determination, and the reason for permitting a repeat test, must be recorded in the subject's source documents and initialed by the investigator. A positive, repeat alcohol or drug screen is exclusionary.

1. Subject has used:
   - - Monoamine oxidase inhibitors (MAOIs) within 12 weeks before screening
     - A known inhibitor or inducer of cytochrome P450 (CYP)3A4 activity (e.g., systemic administration of erythromycin, clarithromycin, ketoconazole, itraconazole, rifampicin) within 14 days or a period less than 5-times the drug’s half-life, whichever is longer, before the first study drug administration on Day 1. Use of moderate and strong inhibitors and inducers of CYP3A4 are prohibited during the study (See Appendix 5).
     - St. John’s wort, ephedra, ginkgo, ginseng, or kava within 2 weeks before screening.
     - Antipsychotic drugs (D_2_-antagonists) within 2 weeks before screening. However, Seroquel (quetiapine) in a dose ≤100 mg is allowed when used in a stable dose for at least 8 weeks prior to screening. Quetiapine treatment should be continued unchanged during the study.
     - Lithium or other mood stabilizers within 2 weeks before screening.
     - Opioids within 2 weeks before screening.
     - Psychostimulants such as methylphenidate or dextroamphetamine within 2 weeks before screening.
     - Psychotropics with antidepressant effects such as atomoxetine or thyroid supplementation, in addition to their SSRI or SNRI treatment within 2 weeks before screening.
     - Proton pump inhibitors within 4 weeks before screening.
2. Subject is unable to stop the following medication from the baseline visit (Visit 2) and throughout the study (tapering during screening period allowed):
   - - Any hypnotics including but not limited to:
       - Benzodiazepines when used only as needed (PRN) are not allowed. A subject may continue to take a benzodiazepine treatment only if:
         - The subject has been taking a stable daily dose for at least 6 weeks prior to screening.
         - The dose does not exceed an equivalent of 2 mg of lorazepam per day.
         - Allowed benzodiazepines are lorazepam (≤2 mg/day), clonazepam (≤0.5 mg/day) and alprazolam (≤1 mg/day) being taken daily. Other benzodiazepines should be discussed before subject enrollment with the study responsible physician.
         - Treatment will be continued unchanged during the study.
       - Sedating antihistamines, including chronic use of diphenhydramine.
       - Continuous use of zolpidem, zoplicon, eszopiclone and ramelteon. Note: Nonbenzodiazepines sleep aids (including: zolpidem, zaleplon, and eszopliclone) are allowed on an as needed (PRN) basis during the study but NOT within 24 hours before being in the clinic and not more than 2 nights in a row.
       - S-adenosyl methionine (SAMe)
       - Melatonin, agomelatine
     - NSAIDs and aspirin.
3. Is unwilling or unable to undergo multiple venipunctures because of poor tolerability or lack of easy access.
4. Is unable to read and understand the ICF and PRO, complete study-related procedures, and/or communicate with the study staff.
5. Subject has any condition for which, in the opinion of the investigator, participation would not be in the best interest of the subject (e.g., compromise the well-being) or that could prevent, limit, or confound the protocol-specified assessments.
6. Subject has had major surgery, (e.g., requiring local or general anesthesia) within 12 weeks before screening, or will not have fully recovered from surgery, or has surgery planned during the time the subject is expected to participate in the study.
7. Is a vulnerable subject (e.g., a person kept in detention).
8. Has received any prior treatment with electroconvulsive therapy, vagal nerve stimulation, or a deep brain stimulation device or treatment with ketamine or esketamine for MDD.
9. Has known allergies, hypersensitivity, or intolerance or any contraindication to any of the excipients of JNJ-67953964 or placebo (refer to IB for JNJ-67953964).
10. Has either donated 1 or more units (approximately 450 mL) of blood or acutely lost an equivalent amount of blood within 60 days before the first dose of study drug.
11. Has cognitive impairment that would render the informed consent invalid or limit the ability of the subject to comply with the study requirements.
12. Subject is an employee of the investigator or study site, with direct involvement in the proposed study or other studies under the direction of that investigator or study site, as well as family members of the employees or the investigator.

NOTE: Investigators should ensure that all study enrollment criteria have been met at screening. If a subject's status changes (including laboratory results) after screening but before first dose of study drug is given such that they now meet an exclusion criterion, they should be excluded from participation in the study.

***Lifestyle Considerations***

Potential subjects must be willing and able to adhere to the following prohibitions and restrictions during the course of the study to be eligible for participation.

1. May not consume food or beverages containing, grapefruit juice, Seville oranges (including any orange marmalade), or quinine (e.g., tonic water) from 24 hours (72 hours in the case of grapefruit juice and Seville oranges) before the first dose of study medication and throughout the duration of the study until the final study visit.
2. Should not take any prohibited medication or food supplements as indicated in the section ‘Concomitant Therapy’.
3. The use of limited amounts of alcohol (up to 2 standard drinks consumptions daily) will be allowed but not within 24 hours before any study visit. A standard drink is defined as: a 350-mL glass of 5% alcohol-by-volume (ABV) beer (1.7 units), a 150-mL glass of 12% ABV wine (2 units), or a 45-mL glass of a 40% ABV (80 proof) spirit (1.7 units).
4. Sleepiness and sedation may be induced by any central action compound. If these adverse effects are noticed by the subject, he/she should not drive a car or operate a machine.
5. Strenuous exercise may affect study specified assessments and laboratory safety results; for this reason, strenuous exercise should be avoided within 24 hours before all planned study visits.
6. May not consume food containing poppy seeds from 72 hours before the screening visit.

**Table S2. Summary of Demographic and Baseline Characteristics (Safety Dataset)**

|  | **Aticaprant 10 mg + SSRI/SNRI n = 85** | **Placebo + SSRI/SNRI n = 84** | **Total N = 169** |
| --- | --- | --- | --- |
| Age (years) |  |  |  |
| Mean (SD) | 43.0 (12.81) | 42.1 (12.54) | 42.6 (12.65) |
| Range | 21 – 64 | 19 – 64 | 19 – 64 |
| Sex, n % |  |  |  |
| Female | 60 (70.6) | 62 (73.8) | 122 (72.2) |
| Male | 25 (29.4) | 22 (26.2) | 47 (27.8) |
| Race, n (%) |  |  |  |
| White | 78 (91.8) | 79 (94.0) | 157 (92.9) |
| Black or African American | 5 (5.9) | 2 (2.4) | 7 (4.1) |
| Asian | 2 (2.4) | 2 (2.4) | 4 (2.4) |
| American Indian or Alaska Native | 0 | 1 (1.2) | 1 (0.6) |
| Country, n (%) |  |  |  |
| Russia | 21 (24.7) | 25 (29.8) | 46 (27.2) |
| United States | 23 (27.1) | 21 (25.0) | 44 (26.0) |
| Moldova | 14 (16.5) | 15 (17.9) | 29 (17.2) |
| United Kingdom | 15 (17.6) | 10 (11.9) | 25 (14.8) |
| Ukraine | 7 (8.2) | 9 (10.7) | 16 (9.5) |
| Germany | 5 (5.9) | 4 (4.8) | 9 (5.3) |
| MADRS total score |  |  |  |
| Mean (SD) | 32.4 (4.22) | 32.7 (4.25) | 32.6 (4.23) |
| Range | 21 – 45 | 26 – 42 | 21 – 45 |
| SHAPS score |  |  |  |
| Mean (SD) | 37.4 (6.16) | 37.7 (6.01) | 37.6 (6.07) |
| Median | 38 | 38 | 38 |
| Range | 14 – 53 | 22 – 55 | 14 – 55 |
| Placebo lead-in responder^a^, n (%) | 23 (27.1) | 22 (26.2) | 45 (26.6) |
| Prior and Ongoing Antidepressants, n (%) |  |  |  |
| SSRI | 66 (77.6) | 65 (77.4) | 131 (77.5) |
| Escitalopram | 24 (28.2) | 28 (33.3) | 52 (30.8) |
| Sertraline | 23 (27.1) | 16 (19.0) | 39 (23.1) |
| Paroxetine | 8 (9.4) | 8 (9.5) | 16 (9.5) |
| Fluoxetine | 6 (7.1) | 7 (8.3) | 13 (7.7) |
| Citalopram | 5 (5.9) | 6 (7.1) | 11 (6.5) |
| SNRI and other agents | 19 (22.4) | 19 (22.6) | 38 (22.5) |
| Venlafaxime | 14 (16.5) | 9 (10.7) | 23 (13.6) |
| Duloxetine | 4 (4.7) | 8 (9.5) | 12 (7.1) |
| Quetiapine | 3 (3.5) | 3 (3.6) | 6 (3.6) |
| Vortioxetine | 0 | 2 (2.4) | 2 (1.2) |
| Desvenlafaxime | 1 (1.2) | 0 | 1 (0.6) |

MADRS = Montgomery-Åsberg Depression Rating Scale; SD = standard deviation; SHAPS = Snaith-Hamilton Pleasure Scale; SNRI = serotonin-norepinephrine reuptake inhibitor; SSRI = selective serotonin reuptake inhibitors.

1. Defined as ≥30% improvement in MADRS total score from baseline.

NOTE: In the eITT dataset, mean (SD) age was 41.6 (12.40) years, 69.4% were female, and mean (SD) MADRS total score and SHAPS score at baseline were 32.9 (4.22) and 38.1 (5.96), respectively.

**Figure S1. Disposition of Participants**


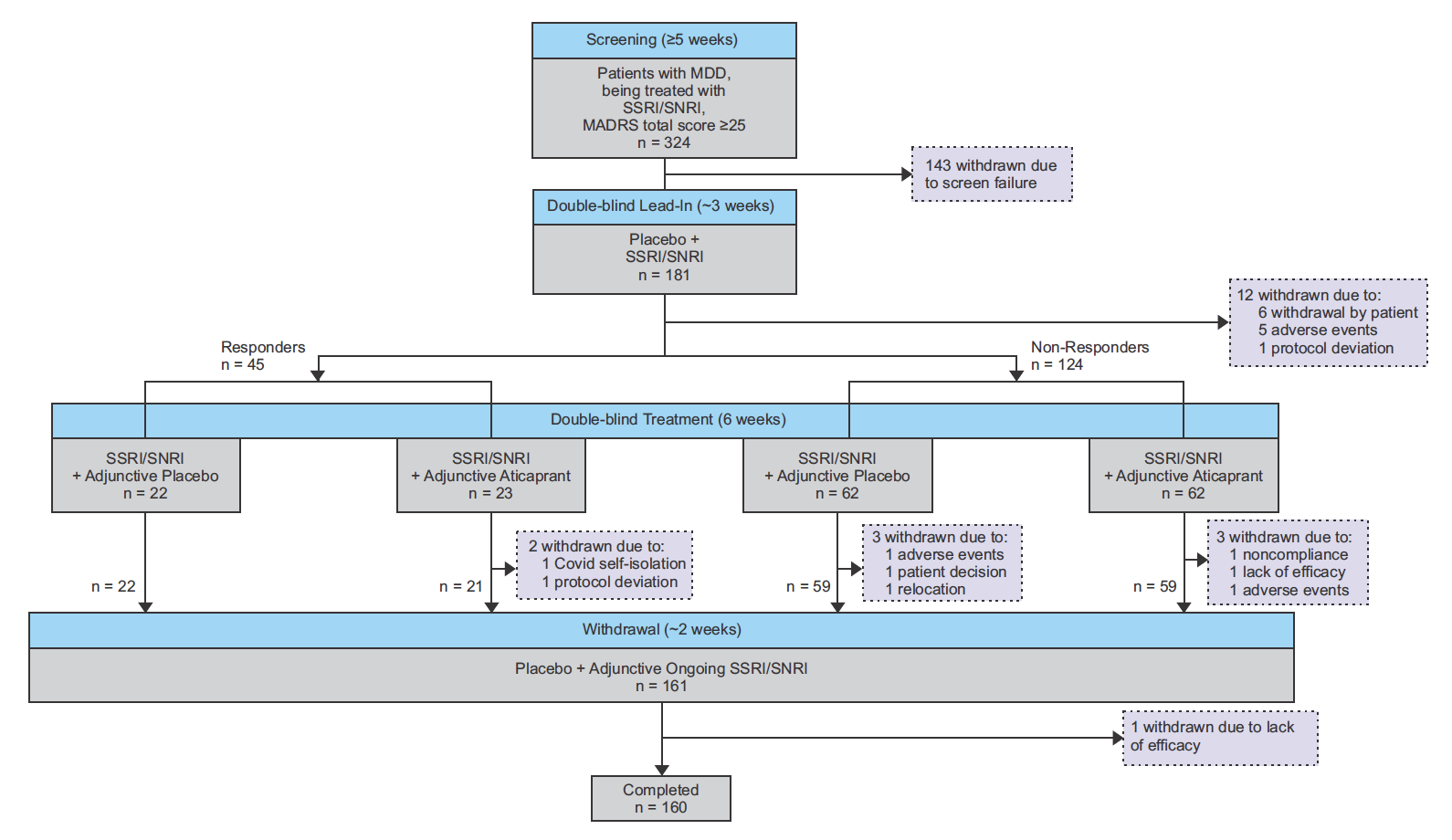


MADRS = Montgomery-Åsberg Depression Rating Scale; MDD = major depressive disorder; SNRI = serotonin-norepinephrine reuptake inhibitor; SSRI = selective serotonin reuptake inhibitor

Note: Placebo responders and placebo non-responders were combined for the full intent-to-treat (fITT) dataset.

**Sensitivity Analyses of the Primary Endpoint**

The impact of COVID-19 on the primary efficacy endpoint was evaluated in sensitivity analyses on all the data collected prior to 15 March 2020 (estimated date of the COVID-19 lockdowns in most of the countries participating in the trial) using the same mixed-effects model repeated measures (MMRM).

Of note, 17% and 19% of the participants in fITT and eITT populations, respectively, had ≥1 MADRS assessment excluded from the MMRM model due to COVID-19. Results of the sensitivity analyses corroborated the findings of the primary efficacy analysis in both the eITT (least squares mean difference [upper limit 1-sided 80% CI]: -3.0 [-1.88]) and fITT (-3.4 [-2.51]) analysis datasets.

**Figure S2. Percentage of Participants in Remission and Percentage Who Were Responders During the Double-Blind Treatment Period (eITT Analysis Dataset)**

1. **Participants in Remission (defined as MADRS total score ≤10)**


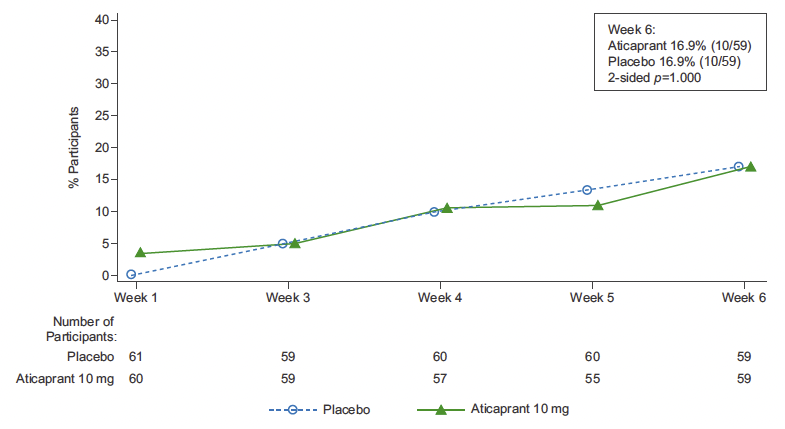


1. **Participants Who Were Responders (defined as ≥30% improvement MADRS total score from treatment baseline)**


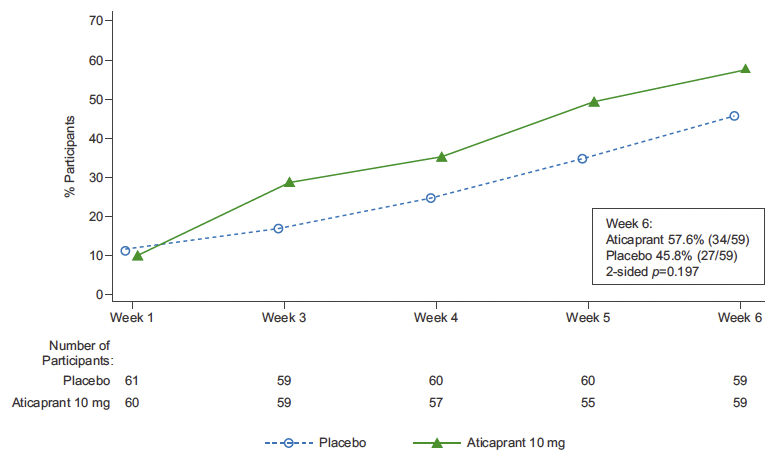


**Figure S2. Percentage of Participants in Remission and Percentage Who Were Responders During the Double-Blind Treatment Period (eITT Analysis Dataset) (continued)**

1. **Participants Who Were Responders (defined as ≥50% improvement MADRS total score from treatment baseline)**

**
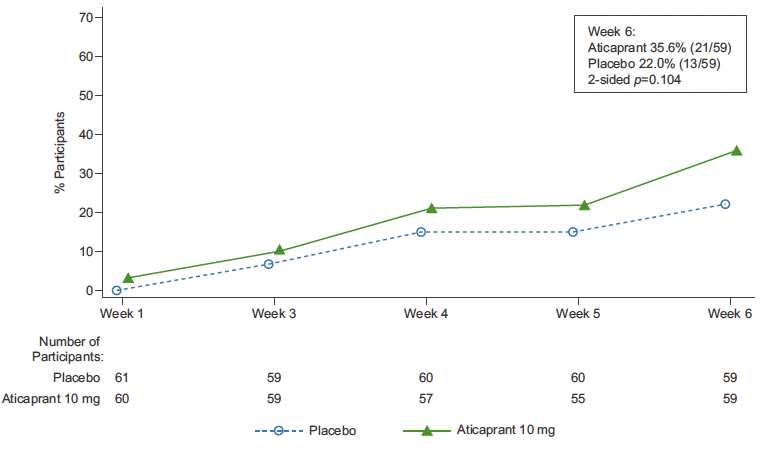
**

eITT = enriched intent-to treat; MADRS = Montgomery-Åsberg Depression Rating Scale

**Figure S3. Percentage of Participants in Remission and Percentage Who Were Responders During the Double-Blind Treatment Period (fITT Analysis Dataset)**

1. **Participants in Remission (defined as MADRS total score ≤10)**


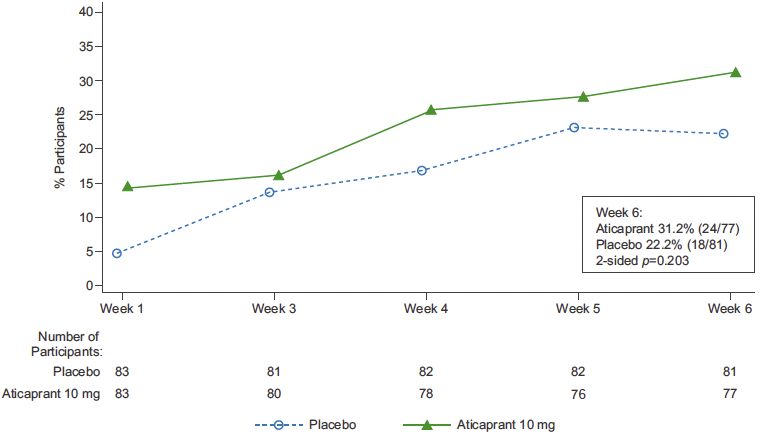


1. **Participants Who Were Responders (defined as ≥30% improvement MADRS total score from treatment baseline)**


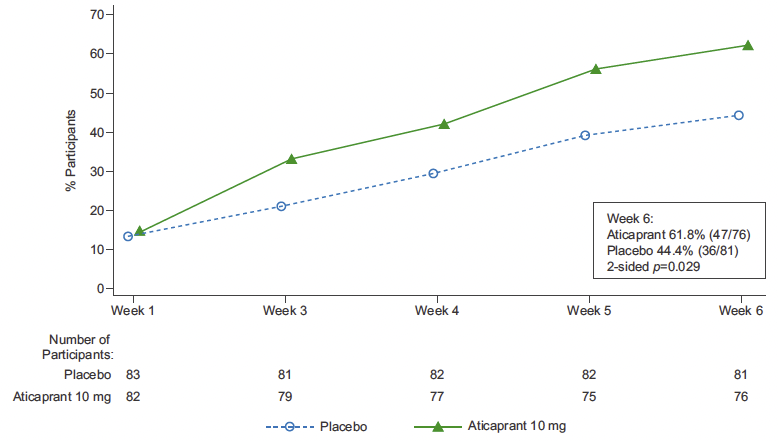


**Figure S3. Percentage of Participants in Remission and Percentage Who Were Responders During the Double-Blind Treatment Period (fITT Analysis Dataset) (continued)**

1. **Participants Who Were Responders (defined as ≥50% improvement MADRS total score from treatment baseline)**

**
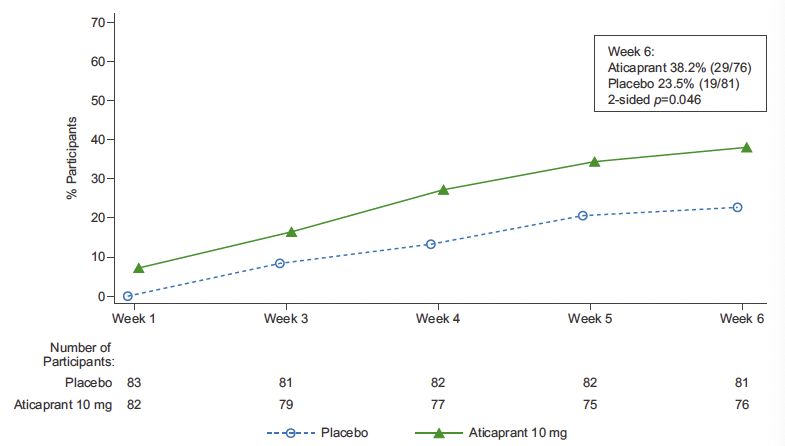
**

fITT = full intent-to treat; MADRS = Montgomery-Åsberg Depression Rating Scale.

**Figure S4. SHAPS Score: LS Mean Change from Baseline (± SE) During Double-Blind Treatment Period**

**
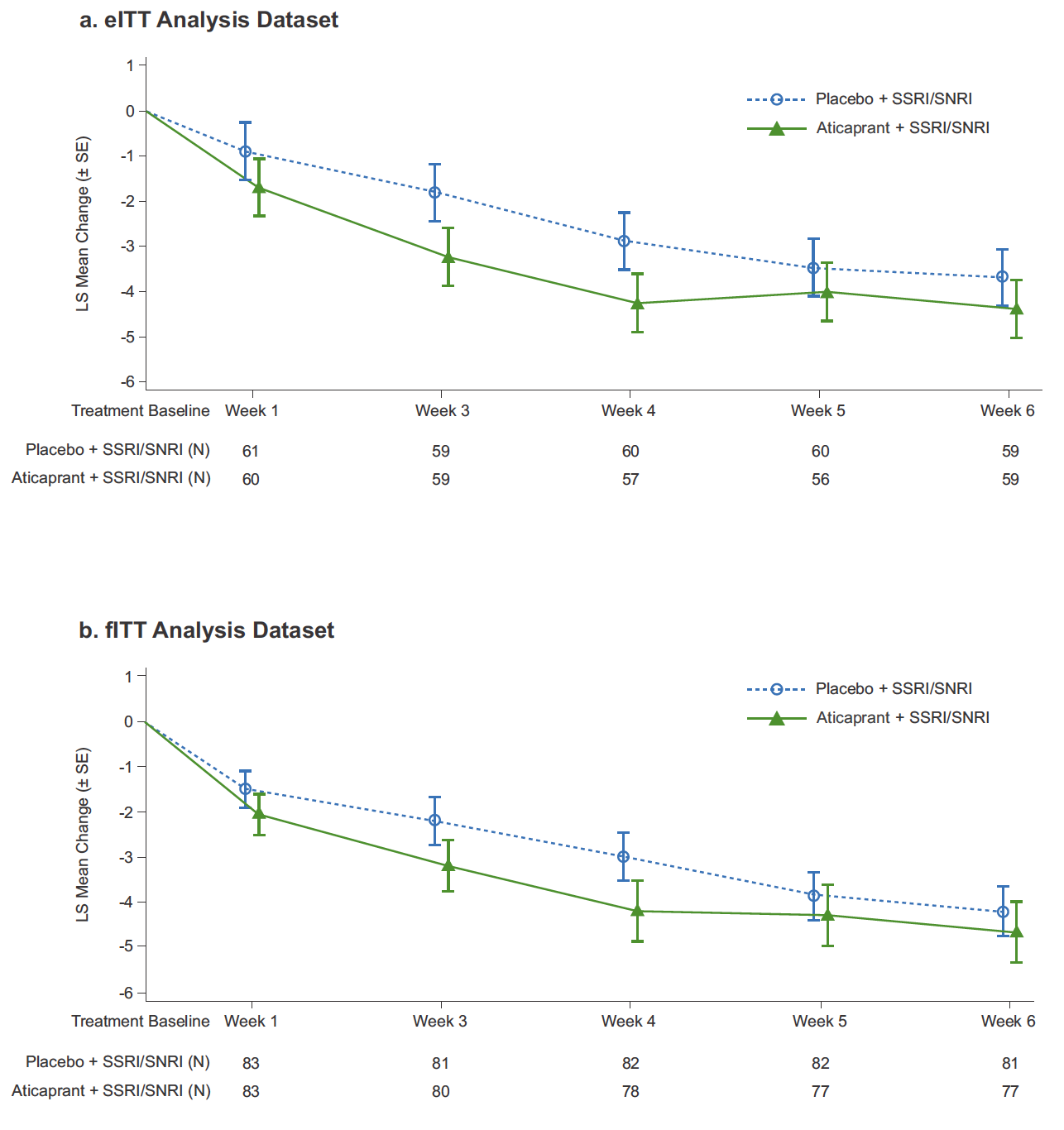
**

eITT = enriched intent-to treat; fITT = full intent-to-treat; LS = least squares; SE = standard error; SHAPS = Snaith-Hamilton Pleasure Scale; SNRI = serotonin-norepinephrine reuptake inhibitor; SSRI = selective serotonin reuptake inhibitor

**Table S3. SHAPS Score: LS Mean Change from Baseline Over 6 Weeks by Level of Anhedonia Severity at Baseline**

|  | **SHAPS Score ≥ Baseline Median** | | **SHAPS Score < Baseline Median** | | |
| --- | --- | --- | --- | --- | --- |
|  | **Aticaprant 10 mg + SSRI/SNRI** | **Placebo +**  **SSRI/SNRI** | **Aticaprant 10 mg + SSRI/SNRI** | **Placebo +**  **SSRI/SNRI** |  |
| **eITT Analysis Dataset** |  |  |  |  |  |
| Baseline |  |  |  |  |  |
| N | 26 | 27 | 34 | 34 |  |
| Mean (SD) | 41.0 (2.78) | 41.7 (3.72) | 32.9 (3.59) | 32.9 (3.71) |  |
| Change to week 6 |  |  |  |  |  |
| N | 26 | 27 | 33 | 32 |  |
| Mean (SD) | -6.85 (7.47) | -5.7 (5.91) | -2.8 (4.37) | -2.8 (3.79) |  |
| MMRM analysis^a^ |  |  |  |  |  |
| Difference of LS means | -1.70 |  | 0.04 |  |  |
| 80% CI on difference | (-3.74, 0.34) |  | (-1.19, 1.27) |  |  |
| 2-sided *p* value | 0.284 |  | 0.966 |  |  |
| **fITT Analysis Dataset** |  |  |  |  |  |
| Baseline |  |  |  |  |  |
| N | 30 | 34 | 53 | 49 |  |
| Mean (SD) | 40.7 (2.74) | 41.3 (3.48) | 31.8 (4.50) | 32.8 (3.51) |  |
| Change to week 6 |  |  |  |  |  |
| N | 29 | 34 | 48 | 47 |  |
| Mean (SD) | -7.1 (7.24) | -5.8 (5.85) | -3.2 (4.38) | -3.1 (3.94) |  |
| MMRM analysis^a^ |  |  |  |  |  |
| Difference of LS means | -2.09 |  | -0.12 |  |  |
| 80% CI on difference | (-3.95, -0.22) |  | (-1.13, 0.89) |  |  |
| 2-sided *p* value | 0.152 |  | 0.878 |  |  |

CI = confidence interval; eITT = enriched intent-to treat; fITT = full intent-to treat; LS = least squares; MMRM = mixed-effects model for repeated measures; SHAPS = Snaith-Hamilton Pleasure Scale; SNRI = serotonin and norepinephrine reuptake inhibitors; SSRI = selective serotonin reuptake inhibitors.

^a^ Test for no difference between treatments from a MMRM model with change from baseline as the response variable, patient as a random effect, time, treatment, country, and time-by-treatment interaction as factors, and baseline value as a covariate.

Notes: SHAPS score ranges from 14-56; a higher score indicates greater severity of anhedonia. Negative change in score indicates improvement. Negative difference favors aticaprant.

**Table S4. MADRS 5-Item Anhedonia Factor Score: LS Mean Change from Baseline Over 6 Weeks by Level of Anhedonia Severity at Baseline**

|  | **SHAPS Score ≥ Baseline Median** | | **SHAPS Score < Baseline Median** | | |
| --- | --- | --- | --- | --- | --- |
|  | **Aticaprant 10 mg + SSRI/SNRI** | **Placebo +**  **SSRI/SNRI** | **Aticaprant 10 mg + SSRI/SNRI** | **Placebo +**  **SSRI/SNRI** |  |
| **eITT Analysis Dataset** |  |  |  |  |  |
| Baseline |  |  |  |  |  |
| N | 26 | 27 | 34 | 34 |  |
| Mean (SD) | 18.6 (2.16) | 19.4 (3.86) | 17.2 (2.52) | 16.1 (3.12) |  |
| Change to week 6 |  |  |  |  |  |
| N | 26 | 27 | 33 | 32 |  |
| Mean (SD) | -6.8 (5.80) | -4.2 (4.70) | -6.0 (4.65) | -5.1 (5.40) |  |
| MMRM analysis^a^ |  |  |  |  |  |
| Difference of LS means (SE) | -2.7 (1.20) |  | -0.4 (1.00) |  |  |
| Upper limit 1-sided 80% CI on difference | -1.64 |  | 0.46 |  |  |
| 1-sided *p* value | 0.015 |  | 0.349 |  |  |
| **fITT Analysis Dataset** |  |  |  |  |  |
| Baseline |  |  |  |  |  |
| N | 30 | 34 | 53 | 49 |  |
| Mean (SD) | 17.8 (3.20) | 18.0 (4.61) | 13.7 (5.72) | 13.7 (4.76) |  |
| Change to week 6 |  |  |  |  |  |
| N | 29 | 34 | 48 | 47 |  |
| Mean (SD) | -6.8 (5.73) | -3.6 (5.05) | -5.5 (4.40) | -4.0 (5.31) |  |
| MMRM analysis^a^ |  |  |  |  |  |
| Difference of LS means (SE) | -3.6 (1.14) |  | -1.2 (0.79) |  |  |
| Upper limit 1-sided 80% CI on difference | -2.59 |  | -0.57 |  |  |
| 1-sided *p* value | 0.001 |  | 0.059 |  |  |

CI = confidence interval; eITT = enriched intent-to treat; fITT = full intent-to treat; LS = least squares; MADRS = Montgomery-Åsberg Depression Rating Scale; MMRM = mixed-effects model for repeated measures; SHAPS = Snaith-Hamilton Pleasure Scale; SNRI = serotonin and norepinephrine reuptake inhibitors; SSRI = selective serotonin reuptake inhibitors.

^a^ Test for no difference between treatments from a MMRM model with change from baseline as the response variable, patient as a random effect, time, treatment, country, and time-by-treatment interaction as factors, and baseline value as a covariate.

Notes: MADRS 5-item anhedonia factor score ranges from 0-30; a higher score indicates a more severe condition. Negative change in score indicates improvement. Negative difference favors aticaprant.

**Table S5. SIGH-A Total Score and HAM-A_6_ Subscale Score: Change from Baseline to Week 6 of Double-Blind Treatment Phase**

|  | **Aticaprant 10 mg + SSRI/SNRI** | **Placebo +**  **SSRI/SNRI** |
| --- | --- | --- |
| **SIGH-A** |  |  |
| **eITT Analysis Dataset** |  |  |
| Baseline |  |  |
| N | 60 | 61 |
| Mean (SD) | 18.8 (5.99) | 19.0 (6.45) |
| Change to week 6 |  |  |
| N | 59 | 59 |
| Mean (SD) | -5.9 (5.37) | -5.4 (6.55) |
| Effect size at week 6 | -0.08 |  |
| MMRM analysis^a^ |  |  |
| Difference of LS means (SE) | -0.7 (0.90) |  |
| 2-sided 80% CI on difference | -1.90, 0.41 |  |
| 2-sided *p* value | 0.410 |  |
| **fITT Analysis Dataset** |  |  |
| Baseline |  |  |
| N | 83 | 83 |
| Mean (SD) | 16.5 (7.10) | 17.1 (6.84) |
| Change to week 6 |  |  |
| N | 77 | 81 |
| Mean (SD) | -5.5 (4.99) | -4.4 (6.19) |
| Effect size at week 6 | -0.20 |  |
| MMRM analysis^a^ |  |  |
| Difference of LS means (SE) | -1.4 (0.73) |  |
| 2-sided 80% CI on difference | -2.31, -0.44 |  |
| 2-sided *p* value | 0.060 |  |

**Table S5. SIGH-A Total Score and HAM-A_6_ Subscale Score: Change from Baseline to Week 6 of Double-Blind Treatment Phase (continued)**

|  | **Aticaprant 10 mg + SSRI/SNRI** | **Placebo +**  **SSRI/SNRI** |
| --- | --- | --- |
| **HAM-A_6_** |  |  |
| **eITT Analysis Dataset** |  |  |
| Baseline |  |  |
| N | 60 | 61 |
| Mean (SD) | 8.4 (2.88) | 8.3 (2.88) |
| Change to week 6 |  |  |
| N | 59 | 59 |
| Mean (SD) | -2.7 (2.65) | -2.2 (2.84) |
| Effect size at week 6 | -0.20 |  |
| MMRM analysis^a^ |  |  |
| Difference of LS means (SE) | -0.6 (0.4) |  |
| 2-sided 80% CI on difference | -1.21, -0.07 |  |
| 2-sided *p* value | 0.148 |  |
| **fITT Analysis Dataset** |  |  |
| Baseline |  |  |
| N | 83 | 83 |
| Mean (SD) | 7.4 (3.39) | 7.5 (3.10) |
| Change to week 6 |  |  |
| N | 77 | 81 |
| Mean (SD) | -2.7 (2.56) | -1.7 (2.84) |
| Effect size at week 6 | -0.37 |  |
| MMRM analysis^a^ |  |  |
| Difference of LS means (SE) | -1.1 (0.37) |  |
| 2-sided 80% CI on difference | -1.56, -0.62 |  |
| 2-sided *p* value | 0.003 |  |

CI = confidence interval; eITT = enriched intent-to treat; fITT = full intent-to treat; HAM-A_6_ = Hamilton Anxiety 6-item subscale; LS = least squares; MMRM = mixed-effects model for repeated measures; SIGH-A = Structured Interview Guide for the Hamilton Anxiety; SNRI = serotonin and norepinephrine reuptake inhibitors; SSRI = selective serotonin reuptake inhibitors

^a^ Test for no difference between treatments from a MMRM model with patient as random effect; subjective sleep disorder, treatment, time, and time-by-treatment interaction as factors; and baseline score (SIGH-A Total Score or HAM-A_6_ subscale score) as continuous covariate. An AR(1) variance-covariance matrix was employed.

Notes: SIGH-A total score ranges from 0-56; HAM-A_6_ score ranges from 0-24; a higher score indicates a more severe condition. Negative change in score indicates improvement. Negative difference favors aticaprant. Negative effect size favors aticaprant.

**Massachusetts General Hospital Cognitive and Physical Function Questionnaire (CPFQ)**

To evaluate the effect of study drug on subjective assessments of cognitive and executive function, participants were asked to complete the Massachusetts General Hospital Cognitive and Physical Function Questionnaire (CPFQ) [Fava et al., 2009]. The CPFQ includes 7 questions about attention, energy, memory, mental acuity, and motivation. It is scored on a 6-point Likert scale, with higher values indicating worse function.

In the eITT analysis dataset, mean (SD [range]) baseline CPFQ total score at treatment baseline was 27.2 (5.67 [14-35]) points for aticaprant and 27.4 (6.04 [15-42]) points for placebo. The mean changes from treatment baseline (SD) in CPFQ total score at week 6 were -4.6 (5.63) and -4.0 (5.16) points for aticaprant and placebo, respectively.

In the fITT analysis dataset, mean (SD [range]) baseline CPFQ total score at treatment baseline was 25.6 (6.37 [7-35]) and 26.0 (6.45 [10-42] for aticaprant and placebo, respectively, and mean changes from treatment baseline (SD) in CPFQ total score at week 6 were -4.2 (5.64) and -3.5 (5.93) points for the respective treatment groups.

The results indicate a numerically greater reduction in CPFQ total scores for aticaprant compared to placebo, but baseline to endpoint changes were not significantly different.

**Reference**

Fava M, Iosifescu DV, Pedrelli P, Baer L. Reliability and validity of the Massachusetts general hospital cognitive and physical functioning questionnaire. Psychother Psychosom. 2009;78(2):91-7. doi: 10.1159/000201934.

**Table S6. Treatment-Emergent Adverse Events During the Placebo Lead-in Period**

|  | **Number (%) of Participants**  **N= 169** |
| --- | --- |
| **Total, Participants with AE** | 55 (32.5) |
| **Adverse Events:** |  |
| Headache | 11 (6.5) |
| Nausea | 7 (4.1) |
| Diarrhea | 6 (3.6) |
| Nasopharyngitis | 6 (3.6) |
| Upper respiratory tract infection | 5 (3.0) |
| Dry mouth | 4 (2.4) |
| Dyspepsia | 3 (1.8) |
| Vomiting | 3 (1.8) |
| Constipation | 2 (1.2) |
| Gastroenteritis viral | 2 (1.2) |
| Hyperhidrosis | 2 (1.2) |
| Migraine | 2 (1.2) |
| Somnolence | 2 (1.2) |

The following adverse events were reported for 1 participant (0.6%) each: abdominal discomfort, abdominal pain, abdominal pain upper, agitation, back pain, benign breast neoplasm, contusion, cystitis, disorientation, dizziness, dyspnea, ear pain, epigastric discomfort, fatigue, flatulence, foot fracture, gastroenteritis, hordeolum, hot flush, hypertension, initial insomnia, micturition disorder, mouth ulceration, muscle strain, myalgia, nasal congestion, oropharyngeal pain, paresthesia, pharyngitis, pruritus, respiratory disorder, rhinorrhea, tooth infection, tinnitus.

**Table S7. Search of Preferred Terms to Identify Adverse Events Suggestive of Abuse Potential**

The following preferred terms were searched to identify adverse events suggestive of abuse potential: aggression, confusional state, decreased activity, dependence, disorientation, dissociation, dissociative disorder, dizziness, drug abuse, drug abuser, drug dependence, drug detoxification, drug diversion, drug rehabilitation, drug tolerance, drug tolerance increased, drug use disorder, drug withdrawal convulsions, drug withdrawal headache, drug withdrawal syndrome, euphoric mood, feeling abnormal, feeling drunk, feeling of relaxation, hallucination, hallucination auditory, hallucination gustatory, hallucination olfactory, hallucination synaesthetic, hallucination tactile, hallucination visual, hallucinations mixed, inappropriate affect, mental impairment, product tampering, psychomotor hyperactivity, psychotic disorder, rebound effect, somatic hallucination, somnolence, substance abuser, substance dependence, substance use, substance use disorder, substance-induced mood disorder, substance-induced psychotic disorder, thinking abnormal, withdrawal arrhythmia, withdrawal syndrome.

**Table S8. Treatment-Emergent Adverse Events During the Withdrawal Period**

|  | **Number (%) of Participants** | |
| --- | --- | --- |
|  | **Aticaprant 10 mg + SSRI/SNRI n = 85** | **Placebo +**  **SSRI/SNRI n = 84** |
| **Total, Participants with AE** | 5 (5.9) | 4 (4.8) |
| **Adverse Events:** |  |  |
| Blood creatine phosphokinase increased | 1 (1.2) | 0 |
| Cough | 0 | 1 (1.2) |
| Dizziness | 0 | 1 (1.2) |
| Headache | 0 | 1 (1.2) |
| Muscle spasms | 1 (1.2) | 0 |
| Muscle strain | 0 | 1 (1.2) |
| Nasopharyngitis | 1 (1.2) | 1 (1.2) |
| Nausea | 0 | 1 (1.2) |
| Oropharyngeal pain | 1 (1.2) | 0 |
| Pulpitis dental | 0 | 1 (1.2) |
| Pyrexia | 1 (1.2) | 0 |
| Respiratory disorder | 1 (1.2) | 0 |
| Rotator cuff syndrome | 0 | 1 (1.2) |

**Other Safety Assessments**
Most hematology and chemistry parameters measured during the double-blind treatment period were within the reference ranges. Elevated glucose was reported in both treatment groups, was present at screening, and the incidence did not change over the study (incidence across visits: 14.6%-21.7% for aticaprant, 15.7%-20.3% for placebo). During the double-blind period, 2 and 5 abnormal laboratory values/associated conditions were reported as an adverse event in the aticaprant group (blood creatine phosphokinase increased, hypoglycemia) and the placebo group (alanine aminotransferase increased, n=2; aspartate aminotransferase increase, blood alkaline phosphatase increased, blood cholesterol increased, n=1 each), respectively.

There were isolated occurrences of vital sign values (blood pressure, pulse, body temperature) outside the normal range during the double-blind treatment period; these were similarly distributed in the aticaprant and placebo groups. None were reported as an adverse event. During the withdrawal period, a low systolic blood pressure value (89 mmHg), not requiring treatment, was measured for 1 participant and mild pyrexia, reported as an adverse event, was measured for 1 participant. Mean change in body weight from baseline to study endpoint (week 11) or early withdrawal is presented in Table S9.

Observations on ECG were unremarkable, with the exception of clinically significant findings for 2 participants, 1 in the placebo lead-in period and the other, in the aticaprant group. The latter participant had abnormal ECG findings at all visits from screening to study endpoint with first degree atrioventricular block reported as a mild adverse event, unrelated to study drug, on days 7 and 30 of the double-blind treatment period.

**Table S9. Body Weight (kg): Mean Change from Baseline to Study Endpoint (Week 11)/Early Withdrawal**

|  | **Aticaprant 10 mg + SSRI/SNRI** | **Placebo +**  **SSRI/SNRI** |
| --- | --- | --- |
| Baseline |  |  |
| N | 82 | 84 |
| Mean (SD) | 78.75 | 76.17 |
| Endpoint/Early Withdrawal |  |  |
| N | 82 | 84 |
| Mean (SD) | 79.02 (15.618) | 76.84 (15.196) |
| Change to week 6 |  |  |
| N | 82 | 84 |
| Mean (SD) | 0.27 (2.008) | 0.67 (4.723) |
